# Patient Perception and Ethical Trade-Offs in Resource Allocation: A Qualitative Study with Conceptual Simulation in a Romanian Municipal Hospital

**DOI:** 10.3390/healthcare14070903

**Published:** 2026-03-31

**Authors:** Andreea-Luiza Palamaru, Carmen Marinela Cumpăt, Mihaela Catalina Vicol, Liviu Oprea, Muthana Zouri, Nicoleta Zouri, Elena Toader

**Affiliations:** 1 Department of Medical Specialties I and III, Grigore T. Popa University of Medicine and Pharmacy, 700115 Iași, Romania; andreea-luiza-e-palamaru@d.umfiasi.ro (A.-L.P.); mihaela.vicol@umfiasi.ro (M.C.V.); liviu.oprea@umfiasi.ro (L.O.); 2 Department of Computer Science, Toronto Metropolitan University, Toronto, ON M5B 2K3, Canada; mzouri@torontomu.ca; 3 School of ASENT3, Centennial College, Toronto, ON M1G 3T8, Canada; nzouri@centennialcollege.ca; 4 Institute of Gastroenterology and Hepatology, St. Spiridon Emergency Clinical Hospital Iaşi, Grigore T. Popa University of Medicine and Pharmacy, 700111 Iași, Romania; elena.toader@umfiasi.ro

**Keywords:** management, medical services, patient satisfaction, simulation modeling, quality of medical care

## Abstract

**Background/Objectives:** Municipal hospitals in transitional health systems operate under structural resource constraints that complicate managerial decision-making and shape patient perceptions. This study examines how patients interpret resource allocation and evaluate the ethical and legitimacy consequences of alternative strategic priorities. **Methods:** A qualitative research design was employed using semi-structured patient interviews. Participants were recruited using purposive sampling based on predefined inclusion criteria: age over 18, hospitalization for digestive symptoms, undergoing diagnostic investigations, and provision of informed consent. Thematic analysis identified key expectation domains related to technological modernization, workforce capacity, infrastructure, and relational communication. These themes were translated into core governance variables and integrated into a conceptual simulation model comparing three allocation scenarios: technological investment, human resource expansion, and status quo preservation. **Results:** Findings show that patient evaluations extend beyond satisfaction to include distributive fairness, symbolic modernization, and institutional legitimacy. Simulation findings suggest that technological investment strengthens symbolic legitimacy and perceived equity but may increase workload and fiscal exposure; workforce expansion enhances relational justice and operational stability yet leaves modernization gaps; and status quo preservation maintains short-term fiscal balance while risking gradual legitimacy erosion. **Conclusions:** The study demonstrates that satisfaction metrics alone are insufficient for governance evaluation. Integrating ethical analysis, organizational legitimacy theory, participatory input, and systems thinking provides a structured framework for assessing resource allocation trade-offs in resource-constrained municipal hospitals.

## 1. Introduction

Municipal hospitals in transitional health systems face persistent structural constraints that shape managerial decision-making and patient experience. In Romania, decentralization reforms have transferred substantial administrative responsibility to local authorities, often without proportional financial and infrastructural support. In this context, local municipal authorities function as an intermediate governance level between the Ministry of Health and individual hospitals.

Consequently, many municipal hospitals function with limited capital budgets, aging infrastructure, workforce migration pressures, and restricted access to advanced diagnostic technologies. Technological concentration in tertiary centers further reinforces disparities in service availability, influencing not only clinical pathways but also public perceptions of institutional fairness and credibility.

Although reforms have aimed to professionalize management and enhance accountability [1], fiscal instability and uneven resource distribution continue to constrain long-term strategic planning [2]. Under such conditions, resource allocation decisions require balancing modernization investments with operational sustainability. Managerial choices regarding technological acquisition, workforce expansion, or fiscal restraint therefore carry distributive, relational, and symbolic implications. Resource scarcity is both an economic constraint and a governance challenge affecting institutional legitimacy.

Allocation decisions in public hospitals are inherently ethical. Distributive justice concerns how limited resources, diagnostic technologies, staffing capacity, infrastructure, are allocated across patient groups and service domains. Access theory emphasizes that availability and accommodation are central to fairness in healthcare delivery [3]. At the same time, opportunity cost considerations are unavoidable: investment in one domain necessarily limits alternatives. Capital-intensive modernization may restrict staffing expansion, while workforce prioritization may delay structural technological upgrades. In systems characterized by structural underinvestment [2], such trade-offs significantly shape equity, quality, and sustainability outcomes.

Managerial decisions do not operate only at a technical or operational level; they also function symbolically. Highly visible investments, such as the acquisition of advanced imaging equipment, signal responsiveness, modernization, and institutional seriousness to patients and the broader community. By contrast, strategic inaction or delayed investment may be perceived as stagnation or lack of commitment. Empirical research in Romania shows that institutional credibility depends not only on clinical performance but also on how stakeholders perceive managerial commitment, transparency, and accountability [4,5]. In this sense, governance visibility becomes an essential component of ethical hospital management. Furthermore, previous qualitative studies in Romanian public hospitals indicate that managerial decisions are shaped by consultative leadership styles, administrative and evidence-based decision models, and broader political and economic constraints [6].

Organizational legitimacy plays a central role in this dynamic. In public hospitals, legitimacy is not determined only by clinical performance, but by the degree to which managerial priorities reflect and align with community expectations. Visible modernization efforts, such as technological acquisitions or infrastructure upgrades, can strengthen perceptions of competence, fairness, and institutional commitment, even when interpersonal satisfaction is already strong. At the same time, legitimacy depends on responsiveness, meaning the extent to which patient concerns are visibly incorporated into strategic planning and decision-making [7]. In resource-constrained health systems [2], legitimacy remains fragile and highly sensitive to how managers handle distributive and relational trade-offs. Research in the Romanian context shows that ethical tensions are especially pronounced under conditions of scarcity, where competing stakeholder interests intensify allocation dilemmas [8].

Existing research on Romanian hospital performance has predominantly emphasized descriptive measures of patient satisfaction [4,5]. While valuable, such approaches do not fully capture the broader ethical and governance consequences of allocation decisions. Recent bibliometric analyses confirm that patient satisfaction research predominantly focuses on measurement techniques rather than governance consequences [9]. The present study advances this literature by introducing a governance-oriented conceptual simulation model grounded in qualitative patient perceptions. Rather than treating perceptions as isolated outcomes, the study defines them within a structured framework to evaluate how alternative strategies, technological investment, workforce expansion, or status quo preservation, produce cascading effects across modernization, equity of access, relational justice, financial sustainability, and perceived legitimacy.

The aim of this study is to explore patient perceptions of hospital management in a Romanian municipal hospital and to simulate the ethical, organizational, and legitimacy consequences of alternative resource allocation strategies under conditions of structural resource scarcity.

## 2. Theoretical Framework

### 2.1. Resource Allocation Ethics

Resource allocation in municipal hospitals is fundamentally normative, particularly in systems characterized by structural underinvestment and fiscal instability. In this study, patient perception is understood as the evaluative judgment patients form regarding how managerial resource allocation decisions reflect fairness, institutional seriousness, and responsiveness under conditions of constraints. In Romania, decentralization reforms have transferred managerial responsibility to local authorities without proportional financial reinforcement, intensifying distributive tensions in everyday governance decisions [1,2]. Empirical analyses of decentralization in Romanian public hospitals demonstrate variable effects on patient satisfaction and institutional performance across time, highlighting the complexity of local managerial autonomy under fiscal constraint [10]. Under such conditions, allocation choices are not merely technical adjustments but ethical determinations concerning priority-setting, fairness, and institutional responsibility.

Distributive justice concerns the equitable allocation of limited diagnostic technologies, staffing resources, and infrastructural investment across patient groups. Access theory emphasizes that availability and accommodation are central dimensions of fairness in healthcare delivery [3]. In municipal settings lacking advanced imaging infrastructure, such as on-site CT capacity, patients may experience structural inequities relative to tertiary centers. Decisions regarding technological acquisition therefore raise questions not only about efficiency but of equal standards of care.

Allocation under scarcity also entails opportunity cost. Investment in capital-intensive modernization may constrain workforce expansion, while prioritizing staffing may delay technological upgrades. This reflects the classical tension between equity and efficiency: maximizing aggregate performance does not necessarily correct structural inequalities, and preserving fiscal sustainability may limit distributive redress. In structurally constrained systems [2], such trade-offs become particularly consequential. Ethical trade-offs refer to the necessary balancing of competing governance priorities, such as equity, modernization, relational justice, and financial sustainability, when allocating limited healthcare resources under structural constraint.

Priority-setting thus constitutes a governance act with ethical and symbolic implications. As demonstrated in the qualitative findings, patients interpret allocation decisions as signals of seriousness, fairness, and responsiveness. These ethical principles directly inform the core governance variables defined in the simulation model, particularly Equity of Access, Financial Sustainability, and Perceived Legitimacy. Resource allocation ethics therefore provides the normative foundation for evaluating alternative strategic scenarios.

### 2.2. Organizational Legitimacy Theory

Organizational legitimacy theory explains how stakeholders evaluate institutional actions as appropriate and credible within a given social context [11]. In public hospitals operating under structural constraints, legitimacy does not derive solely from clinical outcomes but from perceived alignment between managerial priorities and community expectations.

Moral legitimacy is grounded in normative judgment. It reflects evaluations of whether decisions are ethically appropriate and consistent with expectations of fairness and professional responsibility. Moral legitimacy is closely associated with relational justice and equitable access. Patient narratives emphasized dignity, respectful communication, and fairness in diagnostic availability, aligning with ethical accounts of clinical practice [12] and access-based fairness [3].

Pragmatic legitimacy, by contrast, rests on perceived utility and performance. It reflects whether the institution effectively delivers services and responds to practical needs. Staffing adequacy, waiting times, and diagnostic capacity were interpreted by participants as indicators of managerial effectiveness. Empirical studies in Romania similarly demonstrate that trust and satisfaction are shaped by perceived responsiveness and service quality [4,5].

Symbolic legitimacy is reinforced through visible investments. Technological modernization, particularly advanced diagnostic acquisition, functions not only as a functional improvement but as a public signal of competence and institutional seriousness. The qualitative findings identify CT acquisition as a symbolic legitimacy anchor, reinforcing perceptions of parity with better-equipped facilities.

Each allocation strategy therefore generates a distinct legitimacy configuration within the simulation model: technological investment strengthens symbolic legitimacy, workforce expansion enhances moral and pragmatic legitimacy, and strategic inaction risks gradual legitimacy erosion. An integrated legitimacy perspective is thus essential for interpreting how patients assess governance decisions under scarcity.

### 2.3. Participatory Governance

Participatory governance refers to the structured incorporation of stakeholder perspectives into institutional decision-making. In transitional systems marked by decentralization and uneven resource distribution, legitimacy increasingly depends on visible responsiveness to community expectations [2].

Research demonstrates that patient engagement contributes to quality improvement, transparency, and trust formation [13]. In Romania, patient perceptions significantly influence broader evaluations of institutional credibility [4,5]. However, prior research has largely focused on descriptive satisfaction measurement rather than embedding patient-derived priorities into strategic analysis. Earlier hospital-level investigations similarly documented the centrality of interpersonal care and service conditions in shaping patient satisfaction within Romanian emergency units [14].

In this study, participatory governance is defined at the consultative level. Patient narratives are systematically translated into governance variables and parameter weights within the simulation model. Technological modernization, workforce capacity, infrastructure conditions, and relational communication, identified through qualitative analysis, inform the evaluation of allocation strategies. Participation is therefore not symbolic but methodologically integrated into decision modeling.

By incorporating patient expectations into strategic evaluation, the study strengthens governance visibility and enhances interpretive legitimacy. Participatory governance thus functions as both a democratic principle and a structural mechanism for sustaining institutional credibility under resource constraints.

### 2.4. Systems Thinking in Hospital Management

Healthcare organizations function as complex adaptive systems in which interventions produce recursive and interdependent effects. Systems thinking provides the analytical framework necessary to understand how allocation decisions generate feedback loops and non-linear consequences rather than isolated outcomes.

Modernization, staffing, fiscal sustainability, equity, and legitimacy do not operate independently. For example, technological investment may strengthen legitimacy and attract higher patient inflow, which increases workload pressure if workforce capacity remains constant. Conversely, staffing expansion may stabilize operational flow while leaving distributive modernization gaps unresolved. Such reinforcing and balancing loops illustrate that governance effects unfold dynamically over time.

In resource-constrained municipal hospitals, small strategic shifts can produce disproportionate downstream consequences. Visible modernization may increase fiscal exposure, while status quo preservation may gradually erode symbolic credibility. These interactions cannot be captured through linear performance metrics alone.

Systems thinking therefore provides the structural logic linking ethical theory and legitimacy formation to dynamic organizational outcomes. It clarifies that allocation strategies redistribute pressures across domains rather than eliminate them. Integrating systems thinking with resource allocation ethics and legitimacy theory enables a more comprehensive evaluation of governance complexity in municipal hospitals operating under scarcity.

## 3. Materials and Methods

### 3.1. Study Design

This research employed a qualitative exploratory design complemented by a conceptual simulation modeling extension. The primary methodological phase consisted of semi-structured interviews conducted with hospitalized patients in a Romanian municipal hospital. The qualitative component aimed to explore patients’ perceptions of health management, institutional organization, and expectations regarding modernization and service delivery.

Following thematic analysis, the findings were used to parameterize a governance-oriented conceptual simulation model. This hybrid design allowed the study to move beyond descriptive perception analysis toward an evaluative framework examining how different managerial investment strategies may influence institutional legitimacy under resource constraints.

### 3.2. Setting

The study was conducted in the Gastroenterology Department of the Anton Cincu Municipal Hospital in Tecuci, Galaţi County, Romania. The hospital serves an urban/semi-rural population and provides both continuous and outpatient hospitalization services. Available diagnostic resources include ultrasound and digestive endoscopy; however, advanced imaging infrastructure such as Computed Tomography (CT) is not available on-site.

The absence of CT imaging emerged as a recurrent theme in patient interviews and represents a structural limitation characteristic of many Romanian municipal hospitals. These institutions frequently operate under budgetary constraints, uneven distribution of advanced medical technologies, workforce migration pressures, and delayed modernization investments.

### 3.3. Participants and Data Collection

Participants were recruited using purposive sampling based on predefined inclusion criteria to ensure inclusion of individuals with recent direct and recent interaction with hospital services. The study followed a qualitative case study design aimed at analytic rather than statistical generalization. Recruitment continued until thematic saturation was reached, defined as the point at which no substantially new expectation theme emerged from successive interviews. Saturation occurred after twelve interviews, consistent with established qualitative research standards for focused care-based inquiry. Participants were selected based on the following inclusion criteria:

- Age ≥ 18 years;
- Hospitalization for digestive symptoms;
- Underwent diagnostic investigations (functional, imaging, or endoscopic);
- Provided informed consent.

Patients hospitalized for non-digestive conditions or those who declined participation were excluded. Patients’ demographics are presented in Table 1.

Data collection was conducted between October and December 2023. Interviews were performed until theoretical saturation was achieved; no new thematic categories emerged after the tenth interview. Two additional interviews were conducted to confirm saturation. Twelve patients hospitalized for digestive pathology were selected using purposive sampling, consistent with qualitative methodology.

To support the simulation component of the study, a synthetic population of 1000 simulated agents was subsequently generated using Monte Carlo probabilistic resampling with replacement based on the demographic and thematic distributions observed in the 12 participants. This synthetic cohort preserved the empirical proportions of age groups, sex distribution, educational levels, and dominant expectation themes identified in the qualitative sample, without imposing additional distributional assumptions. The synthetic dataset was not used to replace the qualitative findings but to parameterize and test the conceptual governance simulation model.

### 3.4. Thematic Analysis

Interview transcripts were analyzed using reflexive thematic analysis. Audio recordings were transcribed verbatim to ensure accuracy and completeness of the data. The analysis began with line-by-line coding to identify and label meaningful units of text. These initial codes were subsequently grouped into broader categories through thematic clustering and pattern refinement to capture patterns and relationships across interviews. Table 2 presents the code-to-variable mapping framework.

Six analytical dimensions emerged from the coding process: (1) understanding of health management, (2) access to imaging investigations, (3) adequacy of therapeutic resources, (4) relationship with medical staff, (5) perceived quality of services, and (6) expectations regarding managerial priorities. Coding consistency and interpretive coherence were jointly examined by the research team to reduce individual bias and strengthen reliability.

**Figure 1:**
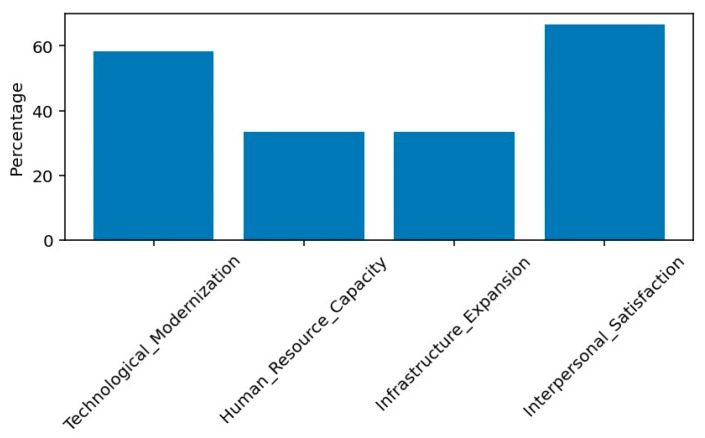
Theme Prevalence.

Four dominant structural expectation themes were identified, each representing distinct yet interconnected dimensions of hospital governance. Technological modernization emerged as a central concern, mentioned by 58.3% of participants (Figure 1). Within this theme, references to “computed” and “tomography” each accounted for 31.8% of theme-specific word frequency, while “CT” represented 18.2%, indicating strong emphasis on diagnostic imaging capacity. Participants framed modernization not only as a clinical necessity but also as a visible signal of institutional progress and managerial competence.

Human resource capacity was identified by 33.3% of participants and represents a complementary operational dimension. Within this theme, “doctor” accounted for 50% of theme-specific references, followed by “physician” and “staff” (each 25%). Respondents emphasized recruitment of additional physicians to improve continuity of care and reduce waiting times. Unlike modernization, which signals progress symbolically, human resources expansion directly affects service delivery efficiency and workload distribution, thereby influencing long-term sustainability of care quality.

Infrastructure expansion was also mentioned by 33.3% of participants, reflecting expectations regarding physical service capacity. The term “hospital” represented 66.7% of theme-specific word frequency, followed by “construction” (22.2%) and “building” (11.1%). These references suggest that participants associate physical space with both dignity of care and institutional stability. Infrastructure therefore operates as a tangible expression of investment priorities and contributes to perceived fairness of resource allocation.

In contrast, interpersonal satisfaction was highly prevalent, reported by 66.7% of participants. The term “relationship” accounted for 100% of word frequency within this theme, underscoring consistently positive evaluations of interactions with medical staff. This dimension represents the relational outcome of governance decisions and serves as an immediate experiential indicator of institutional justice.

The coexistence of high interpersonal satisfaction (66.7%) alongside strong structural reform expectations (58.3% modernization demand) illustrates a dual perception of the institution. While relational trust at the clinical level remains robust, expectations for institutional development persist. This pattern may be described as a “municipal paradox” in local hospital governance: confidence in frontline care coexists with dissatisfaction regarding structural capacity.

Methodological rigor was strengthened through investigator triangulation during thematic coding, with multiple researchers reviewing coded segments to reduce interpretive bias. Initial codes were generated separately and subsequently compared through iterative discussion. Discrepancies were resolved through consensus, and where necessary, a third senior researcher was consulted to ensure consistency and interpretative coherence. Data saturation was confirmed when continued interviewing yielded no new thematic categories. Thematic clusters were further cross tabulated with demographic variables, and frequency analysis was conducted to support qualitative interpretation with descriptive quantitative patterns. The prevalence distribution of major themes is illustrated in Figure 1.

### 3.5. Development of the Conceptual Simulation Model

#### 3.5.1. Derivation of Core Variables

The derivation of the core simulation variables was directly grounded in the thematic structure identified through qualitative analysis. Patient expectations clustered around modernization, staffing capacity, infrastructure conditions, and relational communication. These empirically derived dimensions were translated into governance-relevant constructs to enable structured scenario modeling. The variables were not externally imposed but inductively determined from repeated patterns in patient narratives:

1. Perceived legitimacy was conceptualized as the aggregate judgment patients make regarding the hospital’s managerial credibility, seriousness, and responsiveness to community needs. In the qualitative data, legitimacy was closely associated with visible modernization, institutional progress, and alignment between managerial priorities and patient expectations. Legitimacy captures symbolic, distributive, and relational elements.
2. Patient satisfaction was distinguished from legitimacy and treated as an experiential evaluation of care processes and interpersonal interactions. Interviews revealed generally positive evaluations of doctor-patient relationships. Patient Satisfaction was frequently framed in relation to attentiveness, communication clarity, and responsiveness. Drawing on established quality-of-care frameworks, satisfaction reflects perceived service quality at the point of care rather than structural institutional evaluation [15,16]. In the simulation model, satisfaction is primarily affected by relational justice and staffing capacity but may also be indirectly influenced by modernization through confidence in clinical adequacy.
3. Equity of access emerged prominently in relation to diagnostic limitations, particularly the absence of on-site CT imaging. Participants frequently associate external referrals with structural inequality relative to better-equipped urban hospitals. Therefore, equity of access was defined as the degree to which patients can obtain necessary diagnostic and therapeutic services within the municipal setting without disproportionate burden. This variable reflects distributive justice considerations and aligns with access theory [3]. In the simulation, equity increases under technological investment scenarios and remains moderate or low under staffing-only or status quo strategies.
4. Financial sustainability was derived not from patient perception directly but from the structural context of municipal hospital governance. Romanian municipal hospitals operate under constrained budgets and delayed modernization cycles. Consequently, any strategic investment must be evaluated against fiscal resilience and long-term budgetary equilibrium. Within the simulation, financial sustainability represents the institution’s capacity to maintain operations without generating unsustainable deficit risk. It inversely correlates with capital-intensive modernization and positively correlates with conservative or incremental strategies.
5. The Institutional Modernization Index was constructed as a synthetic indicator reflecting visible technological and infrastructural advancement. Its derivation was directly linked to patient emphasis on CT acquisition as a “symbolic legitimacy anchor”. Modernization was repeatedly framed as a marker of institutional seriousness and progress rather than solely as a technical upgrade. The index therefore captures both material improvement and symbolic signaling effects. In the simulation model, modernization exerts a direct positive effect on perceived legitimacy and equity of access, while indirectly influencing patient inflow and workload dynamics.
6. Relational justice was derived from patient narratives emphasizing empathy, clear explanation, respectful dialog, and moral engagement. Unlike satisfaction, which reflects evaluative response to service quality, relational justice represents a normative expectation of dignity and fairness within interpersonal encounters. This construct aligns conceptually with the ethical foundations of clinical medicine and with literature linking communication quality to trust formation [12]. In the simulation framework, relational justice is primarily strengthened through workforce expansion and improved availability, and it directly contributes to patient satisfaction and legitimacy formation.

These six constructs represent how patients translate structural conditions into institutional judgements. The simulation model was developed as a structured extension of the qualitative findings. Parameter weights were assigned based on thematic frequency, emphasis, and normative salience identified during analysis. Higher weight was attributed to domains that emerged as both recurrent and ethically central in patient narratives, particularly relational justice and modernization. Weights were normalized to sum to one. Please see Table 3.

To enhance conceptual clarity and transparency, Table 4 presents the definitions of the core governance variables and the ethical profile outcome, together with their theoretical grounding

Scenario-specific values were assigned in a structured and theory-informed manner, guided by thematic findings, normative salience, and expected directional relationships between governance domains.

#### 3.5.2. Scenario Construction

The strategic scenarios were constructed by translating the dominant qualitative expectation clusters into distinct managerial allocation pathways under conditions of structural scarcity. Each scenario represents a distinct prioritization strategy grounded in the empirically derived governance variables, perceived legitimacy, patient satisfaction, equity of access, financial sustainability, institutional modernization, and relational justice. The scenarios were not designed as theoretical extremes but as realistic strategic options available to Romanian municipal hospitals operating within budgetary constraints and uneven technological distribution [1,2].

##### Scenario 1—Technological Investment Strategy

The Technological Investment Strategy prioritizes acquisition of a Computed Tomography (CT) scanner and expansion of diagnostic infrastructure. This orientation directly reflects the strongest symbolic expectation identified in qualitative analysis: modernization as a visible marker of institutional seriousness and professional adequacy.

Within established access theory, structural availability of diagnostic services constitutes a core dimension of equitable healthcare provision [3]. The absence of CT imaging in the municipal setting was repeatedly associated with external referrals and perceived inequality relative to larger facilities. Accordingly, this scenario is expected to produce a substantial increase in the Institutional Modernization Index and equity of access.

From a perceived legitimacy perspective, visible technological acquisition functions as a symbolic signal of responsiveness and distributive justice. Organizational legitimacy theory suggests that public institutions strengthen credibility when managerial actions align with community expectations. In this case, CT acquisition represents such alignment.

However, modernization is capital-intensive. In the simulation, this is reflected by a reduction in Financial Sustainability (F = 0.7), indicating increased financial pressure associated with fixed investment costs. This trade-off reflects a classical tension between distributive investment and fiscal prudence. At the same time, modernization is substantially increased (M = 1.5) and equity of access improved (E = 1.4), enhancing perceived service capacity and institutional attractiveness. As suggested in health systems literature, such improvements can increase patient inflow, thereby amplifying workload pressure when staffing levels remain constant [17,18]. This dynamic is captured in the model through a time-dependent decline in Relational Justice, reflecting reduced interpersonal capacity under rising demand. Thus, while this scenario maximizes symbolic and distributive legitimacy, it introduces operational and financial risk.

##### Scenario 2—Human Resource Strategy

The Human Resource Strategy prioritizes recruitment of physicians and support staff. In the simulation, this scenario is operationalized through a positive adjustment in relational and experiential dimensions, with Relational Justice increasing (Rt = baseline trust + 0.2) and Perceived Legitimacy Feedback also strengthening (P = 1.2), reflecting improved communication, responsiveness, and interpersonal engagement. This directly responds to patient concerns regarding insufficient personnel, waiting times, and relational attentiveness. Unlike technological modernization, staffing expansion targets procedural fairness and interpersonal engagement.

The literature on quality of care emphasizes that patient satisfaction is strongly associated with communication quality, responsiveness, and perceived attentiveness [15,16,19]. In this context, workforce expansion strengthens relational justice and directly enhances experiential satisfaction. Furthermore, increased nurse and physician staffing levels have been linked to patient safety and operational efficiency [17,18], supporting the expectation that additional personnel stabilize service flow and reduce workload strain.

However, because diagnostic infrastructure remains unchanged, structural imaging inequality persists. In the simulation, modernization remains stable (M = 1.0), while equity of access improves only marginally (E = 1.1), reflecting indirect gains through improved service flow rather than structural technological enhancement. As a result, distributive inequalities associated with limited diagnostic capacity are not fully resolved. Symbolic modernization remains moderate, as no visible technological advancement is introduced.

From a financial perspective, this strategy maintains relatively stronger fiscal balance compared to capital-intensive modernization, although some cost pressure is introduced through staffing expansion (F = 0.9). Ethically, this scenario prioritizes relational justice and procedural fairness over distributive technological equity. It represents a governance model focused on strengthening moral and interpersonal dimensions of care [12], while maintaining comparatively stronger financial sustainability than capital-intensive modernization.

##### Scenario 3—Status Quo Strategy

The Status Quo Strategy maintains current operational arrangements and prioritizes fiscal stability. In the simulation, this scenario is represented by baseline or slightly declining values across governance domains, with no major technological acquisition or workforce expansion undertaken. Specifically financial sustainability remains stable at a high level (F = 1.0), reflecting minimal additional expenditure, while modernization is reduced (M = 0.8) and equity of access remains limited (E = 0.9), capturing the absence of structural investment in diagnostic capacity. This approach reflects a conservative managerial orientation under constrained public budgets [2].

Relational dynamics are modeled as gradually declining over time (Rt = baseline trust − 0.05 × t), reflecting stagnation in staffing capacity and increasing strain on interpersonal engagement. Similarly, Perceived Legitimacy Feedback is reduced (P = 0.9), capturing the cumulative effect of limited responsiveness to patient expectations. In the short term, workload pressure remains relatively stable due to the absence of demand amplification; however, no improvements in service capacity or relational responsiveness are achieved.

Patient perception studies in Romania and other healthcare systems demonstrate that satisfaction and institutional credibility are shaped not only by immediate interpersonal encounters but also by broader perceptions of system quality and modernization [4,5]. In this context, the absence of visible investment leads to stagnation in symbolic and distributive legitimacy. Over time, this produces a gradual erosion of institutional credibility, even if short-term satisfaction remains acceptable.

This scenario embodies a governance trade-off in which fiscal prudence is preserved (F = 1.0) at the cost of declining relational and legitimacy dynamics and persistent structural limitations (M = 0.8; E = 0.9). It reflects stability without reform, where short-term equilibrium is maintained but long-term institutional competitiveness and credibility are progressively weakened.

#### 3.5.3. Simulation Tools

To define the qualitative findings within a structured evaluative framework, three complementary simulation tools were employed: (A) decision tree modeling, (B) a system dynamics framework, and (C) a multi-criteria ethical evaluation grid.

1. Decision Tree Modeling

A decision tree modeling approach was selected because the research problem involves discrete managerial choices followed by logically ordered and conditionally dependent consequences. In the context of resource allocation, hospital leadership must select one strategic option among mutually exclusive alternatives, and each option produces a structured sequence of operational and institutional implications. Decision trees are particularly appropriate for modeling such scenarios because they provide a transparent representation of branching pathways, clarify conditional relationships between decisions and outcomes, and allow systematic comparison of strategic trade-offs across predefined domains (e.g., modernization, staffing capacity, legitimacy, patient satisfaction, equity, workload, and financial sustainability). When applied to the qualitative findings, the decision tree clarifies how alternative strategic priorities generate distinct institutional trajectories, making explicit operational, relational, and financial consequences of each resource allocation pathway.

2. Systems Dynamics Framework

Because hospital governance involves non-linear interactions, a systems dynamics framework was incorporated to model reinforcing and balancing feedback loops. This approach captures demand amplification effects under technological expansion, workload stabilization under workforce growth, and potential legitimacy erosion under prolonged strategic inaction. By modeling these feedback processes over time, the framework demonstrates how initial strategic gains may generate secondary effects, including workload and burnout, thereby influencing long-term institutional stability. In this way, the system dynamics model complements the decision tree by revealing the temporal and systemic consequences of alternate resource allocation strategies rather than limiting the analysis to static decision pathways.

3. Multi-Criteria Ethical Evaluation Grid

To integrate empirical findings with normative assessment, a multi-criteria ethical evaluation grid was constructed. This approach is appropriate because hospital resource allocation involves competing values, such as equity, efficiency, legitimacy, transparency, and relational justice, that cannot be reduced to a single performance metric. The grid structure enables the explicit identification and operationalization of these criteria, allowing each strategic option to be evaluated comparatively against a consistent ethical framework. By organizing evaluation across predefined domains, the method enhances transparency in normative judgment, reduces implicit bias in interpretation, and facilitates structured comparison of trade-offs.

### 3.6. Analytical Approach

The study adopted a conceptual simulation framework informed by qualitative-derived parameter weighting. Interview findings guided both the structure of the model and the relative importance assigned to five governance domains: Relational Justice (*R*), Institutional Modernization (*M*), Equity of Access (*E*), Financial Sustainability (*F*), and Perceived Legitimacy Feedback (*P*).

Institutional legitimacy at time *t* was modeled as a weighted linear function:

*L*_*t*_ = *αR_t_* + *βM_t_* + *γE_t_* + *δF_t_* + *θP_t_* (1)

where *α*, *β*, *γ*, *δ*, and *θ* represent normalized parameter weights such that:

*α* + *β* + *γ* + *δ* + *θ* = 1. (2)

Weights were derived from thematic frequency, intensity of emphasis, and normative salience observed in the qualitative analysis. Based on the prominence of relational satisfaction and modernization demands, the baseline specification assigned *α* = 0.30, *β* = 0.25, *γ* = 0.20, *δ* = 0.15, and *θ* = 0.10.

Governance variables were operationalized as normalized continuous indices, allowing comparability across domains. The resulting composite legitimacy scores were calculated as weighted sums of these variables and expressed on an aggregated index scale (approximately 0–10), where higher values indicate stronger governance performance under each scenario.

These parameters were applied across alternative managerial scenarios to generate comparative governance scores. The model was not intended to produce statistical forecasts; rather, it provided a structured evaluation of how resource allocation strategies redistribute ethical and operational trade-offs under fiscal constraints.

Robustness was assessed through sensitivity analysis, varying weights within a ±15% range while maintaining normalization. Scenario rankings remained stable across plausible parameter perturbations, supporting the internal consistency of the analytical framework.

## 4. Results

### 4.1. Qualitative Findings: Patient Expectation Theme

Thematic analysis of the interview dataset identified four dominant expectation themes across participants. These themes represent structured patient perceptions of managerial responsibility under resource constraints. Rather than isolated complaints, responses revealed patterned expectations tied to governance legitimacy and institutional priorities.

The four dominant themes were:

- Technological modernization.
- Human resource capacity.
- Infrastructure modernization.
- Communication and relational care.

These four dimensions do not represent isolated service preferences but structured governance expectations that shape how patients evaluate institutional legitimacy under constrained resource conditions. Each dimension corresponds to a distinct pathway through which managerial decisions influence trust, perceived fairness, and long-term institutional stability.

The most symbolically salient theme identified in the dataset concerned the absence of advanced diagnostic equipment, particularly CT imaging capacity. Participants repeatedly referred to the need for modern diagnostic technology as a marker of institutional credibility and clinical seriousness. In their accounts, technological capacity functioned not merely as a technical enhancement but as a visible representation of the hospital’s overall standing and competence.

Technology was framed as a signal of modernization, a prerequisite for equitable access to care, and an indicator of managerial effectiveness. Several respondents linked limited diagnostic capacity to delays in care, referrals to external facilities, and a perceived inferiority relative to larger urban hospitals. Within this narrative structure, the CT scanner emerged as a symbolic legitimacy anchor, simultaneously representing clinical adequacy and institutional dignity.

The qualitative tone suggests that technological acquisition would increase perceived modernization, strengthen trust in clinical competence, and reduce diagnostic inequality. Consequently, technological investment was interpreted not only as an efficiency-enhancing intervention but as a justice-related expectation connected to equal standards of care and the moral responsibility of public hospital governance. When followed as a primary strategy, technological modernization strengthens symbolic and distributive legitimacy and reduces perceived diagnostic inequality. However, absent parallel staffing reinforcement, increased institutional credibility may amplify demand and workload pressure, potentially destabilizing relational quality over time.

The second dominant expectation theme concerned staffing levels and workforce renewal. Across interviews, participants repeatedly referenced insufficient personnel, overextended medical staff, prolonged waiting times, and concerns suggestive of professional burnout. In contrast to technological modernization, which was described in symbolic and structural terms, staffing deficiencies were framed primarily as relational and operational problems. Respondents emphasized the human dimension of care, linking staff shortages to reduced attentiveness, fragmented communication, and delayed clinical interventions.

Importantly, staffing adequacy was not conceptualized merely as a numerical increase in personnel. Rather, participants associated workforce capacity with availability, responsiveness, and the quality of interpersonal engagement. Several narratives implicitly connected adequate staffing to patient dignity and moral recognition, suggesting that timely interaction and attentive communication are perceived as indicators of respect and institutional accountability.

This theme therefore reflects heightened sensitivity to relational justice, procedural fairness, and perceived respect within the care process. While staff recruitment was viewed as likely to improve service throughput and interpersonal care quality, participants did not interpret it as a solution to structural diagnostic limitations. Instead, workforce expansion was understood as strengthening the relational and operational integrity of the hospital, particularly in the domains of communication, timeliness, and perceived fairness of treatment. When prioritized, workforce expansion improves relational justice, stabilizes patient satisfaction, and mitigates burnout-related legitimacy erosion. However, staffing alone does not resolve structural diagnostic limitations, leaving distributive equity concerns partially unaddressed.

The third expectation theme concerned hospital infrastructure and the physical care environment. Participants referred to building conditions, cleanliness, spatial adequacy, and the comfort and modernization of patient rooms. Although this theme did not carry the same symbolic weight as advanced diagnostic equipment acquisition, infrastructure was consistently framed as shaping patient comfort and perceptions of institutional investment.

Interview responses indicate that environmental quality functions as a visible indicator of organizational commitment. Clean and well-maintained facilities were associated with institutional seriousness, community pride, and confidence in safety standards. Participants implicitly linked the physical environment to broader judgments about how the hospital is managed and whether it reflects care, responsibility, and attention to public needs.

Infrastructure modernization was therefore interpreted as signaling governance engagement and responsiveness. However, unlike technological investment, it was not directly associated with improvements in diagnostic capacity or clinical performance. Rather, this theme represents a middle-layer legitimacy mechanism: environmental conditions influence institutional image and perceived care quality, strengthening symbolic and pragmatic legitimacy, yet they do not independently address structural clinical constraints. Infrastructure modernization therefore functions as an intermediate legitimacy mechanism: it enhances institutional image and signals managerial engagement yet produces limited direct improvement in clinical equity or workload dynamics when implemented independently.

The fourth expectation theme concerned communication, empathy, and the explanation of medical decisions. Participants emphasized the importance of clear explanations regarding diagnosis and treatment, direct interaction with physicians, emotional reassurance, and a respectful tone throughout the care process.

Unlike staffing levels, which reflect structural capacity and operational resources, communication was framed as a matter of moral engagement. Relational care expectations were frequently articulated in normative terms, suggesting that patients perceive communication as an optional courtesy but as an intrinsic component of legitimate care. Explanatory transparency was repeatedly linked to trust, while empathy and respectful dialog were associated with fairness and recognition.

This theme aligns closely with relational justice and moral legitimacy. Notably, expectations regarding communication were expressed even in cases where participants reported satisfaction with clinical outcomes. This pattern indicates that legitimacy judgments extend beyond medical effectiveness and encompass the interpersonal and ethical dimensions of governance. In this sense, communication functions as a core legitimacy mechanism through which managerial priorities are evaluated at the point of care. Strengthening communication and empathy directly reinforces relational justice and trust formation, producing immediate gains in perceived legitimacy even under material constraint. However, communication cannot sustainably compensate for structural deficiencies in diagnostic capacity or staffing adequacy.

Across the dataset, patient expectations did not appear as isolated or incidental preferences but rather as components of a structured hierarchy of managerial responsibility. Technological investment was associated with symbolic and distributive legitimacy, reflecting concerns about modernization and equitable access to diagnostic services. Staffing capacity was linked to operational fairness and responsiveness, emphasizing timely care and interpersonal engagement. Infrastructure quality functioned as environmental signaling of institutional commitment, shaping perceptions of seriousness and accountability. Communication and empathy, in turn, were grounded in moral recognition and relational justice, reflecting expectations of dignity and respectful treatment.

The four dimensions reveal that municipal hospital governance operated within multidimensional ethical balancing rather than single-variable optimization. The qualitative results indicate that no single dimension independently secures stable legitimacy; instead, institutional credibility depends on how modernization, workforce capacity, infrastructure quality, and relational engagement are integrated. This insight directly motivated the subsequent simulation modeling of alternative allocation strategies.

The qualitative findings therefore indicate that patients assess hospital governance through multidimensional criteria that extend beyond conventional satisfaction measures. Rather than focusing solely on service outcomes, participants articulated expectations tied to justice, legitimacy, and visible managerial priorities. These structured expectations constitute the empirical foundation for the subsequent conceptual simulation modeling of alternative resource allocation strategies.

### 4.2. Simulation Outcomes

Simulation modeling was conducted using qualitative-derived parameter weights distributed across five governance domains: perceived legitimacy, patient satisfaction, equity of access, financial sustainability, institutional modernization, relational justice. These weights were applied to generate structured, comparative scores for three alternative managerial allocation strategies. The simulation parameters were informed by interview responses that shaped thematic prioritization within the model.

The resulting weighted governance scores demonstrate a clear hierarchy of strategic performance. Technological Strategy achieved the highest overall score (7.3), followed by the Human Resource Strategy (6.6), while the Status Quo scenario ranked substantially lower (4.1). Sensitivity analysis, conducted by increasing the modernization weight by 10%, did not alter the ranking order, confirming the relative stability and internal consistency of the model.

Workload dynamics were evaluated using a simplified systems feedback index designed to capture demand amplification effects. The Technological Strategy generated the highest positive workload pressure value (+2.9), indicating increased systemic strain associated with demand growth. In contrast, the Human Resource Strategy produced negative index value (−1.5), suggesting that expanded staffing capacity buffers demand and stabilize operational flow. The Status Quo scenario showed a modest negative value (−0.7), reflecting short-term workload balance in the absence of increased demand. Positive index values indicate rising workload pressure, whereas negative values reflect relative staffing equilibrium.

#### 4.2.1. Scenario 1—Technological Strategy

**Figure 2:**
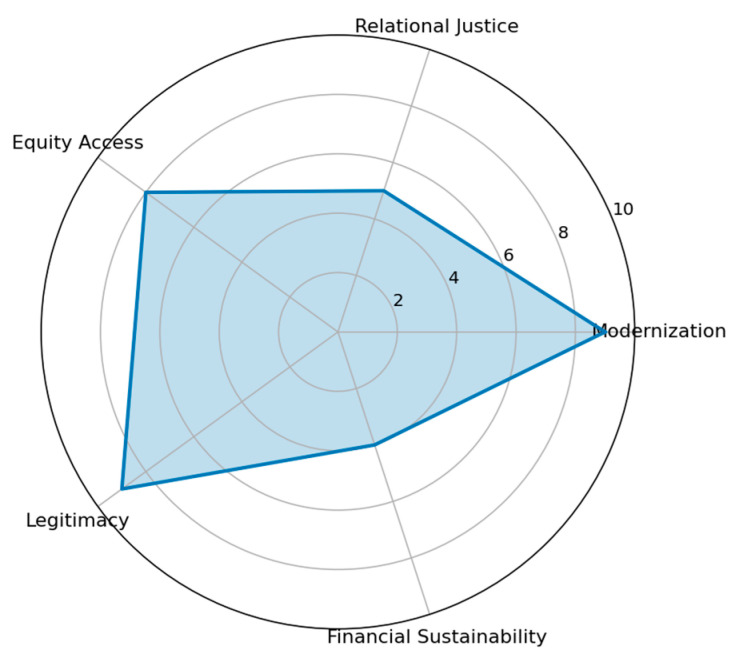
Ethical Profile—Technology Strategy.

The Technological Strategy achieved the highest overall weighted governance score (7.3), indicating superior aggregate performance across the five modeled domains. As illustrated in Figure 2, the radar configuration displays peak values in modernization and legitimacy, alongside strong performance in equity of access. When this strategic pathway is pursued, the immediate consequence is a substantial increase in perceived legitimacy, largely driven by visible technological advancement and the reduction in diagnostic inequality. This pattern is consistent with qualitative findings identifying CT acquisition as a symbolic legitimacy anchor within the hospital governance structure.

The strategy generated the strongest Institutional Modernization Index and the highest perceived legitimacy score among the three scenarios. Visible technological acquisition functions as a powerful signal of institutional seriousness and distributive justice, directly addressing patient concerns regarding diagnostic adequacy and perceived inferiority relative to larger facilities. Improvements in imaging capacity significantly enhance equity of access, reducing structural diagnostic inequality highlighted in the interviews.

Sensitivity analysis further confirmed the robustness of this outcome. When the modernization weight was increased by 10%, the Technological Strategy retained its leading position (modified score: 7.34), indicating that its dominance is not an artifact of parameter selection but reflects structural advantages within the model.

Despite its symbolic and distributive gains, the Technological Strategy exhibits comparatively weaker performance in financial sustainability. Capital-intensive investment introduces fiscal pressure, lowering overall budget stability relative to alternative strategies. This trade-off underscores a core governance dilemma: visible modernization enhances legitimacy but increases medium-term financial exposure unless accompanied by compensatory adjustments in revenue streams or operational efficiency.

**Figure 3:**
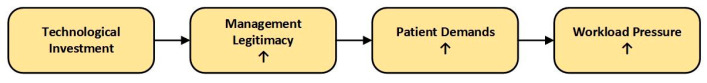
Technological Investment as a Demand-Amplifying Feedback Loop.

The systems feedback index produced the highest workload pressure value (+2.9), indicating demand amplification effects. Modernization strengthens legitimacy and attracts increased patient inflow; however, in the absence of proportional staffing expansion, this generates escalating operational strain. The simulation therefore confirms the hypothesized feedback loop embedded in the systems model (see Figure 3):

Therefore, while the Technological Strategy maximizes symbolic and distributive legitimacy, it simultaneously introduces operational stress and fiscal risk. Its governance advantage is therefore conditional upon parallel adjustments in workforce capacity and financial planning.

#### 4.2.2. Scenario 2—Human Resource Strategy

**Figure 4:**
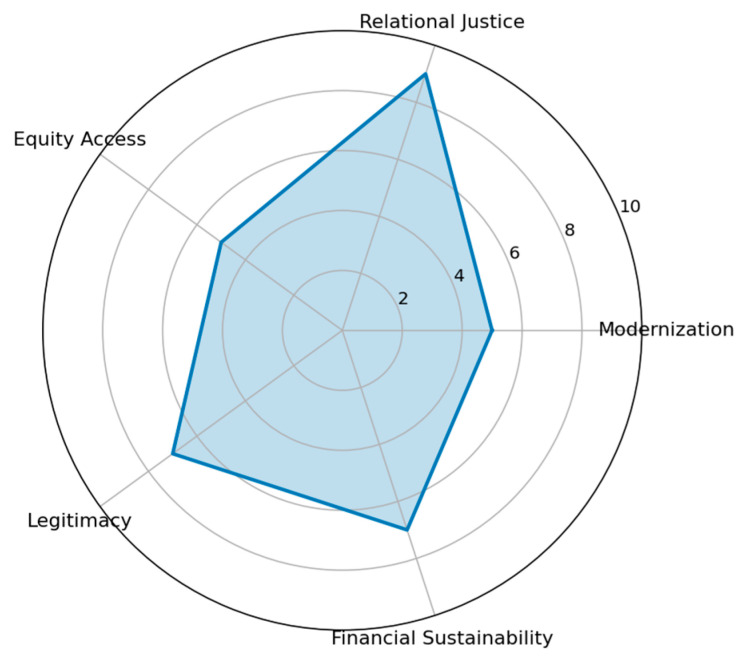
Ethical Profile—Human Resource Strategy.

The Human Resource Strategy achieved an overall weighted governance score of 6.6, ranking second but remaining relatively close to the Technological Strategy. As shown in Figure 4, the radar profile is characterized by a pronounced peak in relational justice and comparatively balanced performance across financial sustainability and legitimacy, while modernization and equity dimensions remain moderate.

This scenario produces the highest relational justice score among all modeled strategies. Expansion of staffing capacity enhances availability, responsiveness, and interpersonal engagement, directly addressing patient expectations regarding attentiveness and respectful communication identified in the qualitative analysis.

The systems feedback index yielded a negative workload pressure value (−1.5), indicating that additional staffing capacity buffers demand rather than amplifying operational strain. Unlike the Technological Strategy, which generates demand-driven workload escalation, the Human Resource Strategy stabilizes service flow. Increased personnel capacity improves operational throughput, reduces waiting times, and enhances process efficiency, thereby strengthening procedural fairness and perceived dignity within the care process.

These improvements align closely with patient-derived priorities emphasizing empathy, accessibility, and responsiveness as central components of legitimate governance.

Despite its strengths in relational domains, modernization and equity of access remain moderate under this strategy. Because diagnostic infrastructure is not expanded, structural imaging inequities persist. While legitimacy improves through enhanced interpersonal quality, it does not achieve the symbolic intensity observed in the Technological Strategy, where visible modernization serves as a strong institutional signal.

Therefore, the Human Resource Strategy prioritizes relational justice and operational balance but leaves distributive modernization gaps unresolved. Its governance profile is therefore ethically strong in interpersonal and procedural domains yet structurally limited in addressing technological disparities.

**Figure 5:**
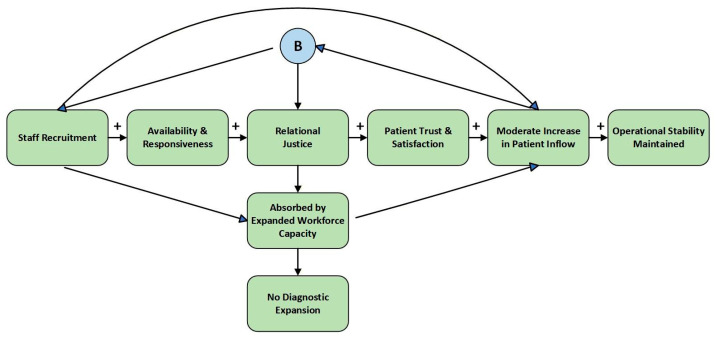
Workforce Expansion as a Demand-Stabilizing Feedback Loop.

Figure 5 shows how workforce expansion absorbs moderate increases in patient inflow rather than amplifying workload strains. Also, it demonstrates that improvements in relational justice generate trust and patient inflow, yet expanded capacity stabilizes demand and maintains operational equilibrium.

#### 4.2.3. Status Quo

**Figure 6:**
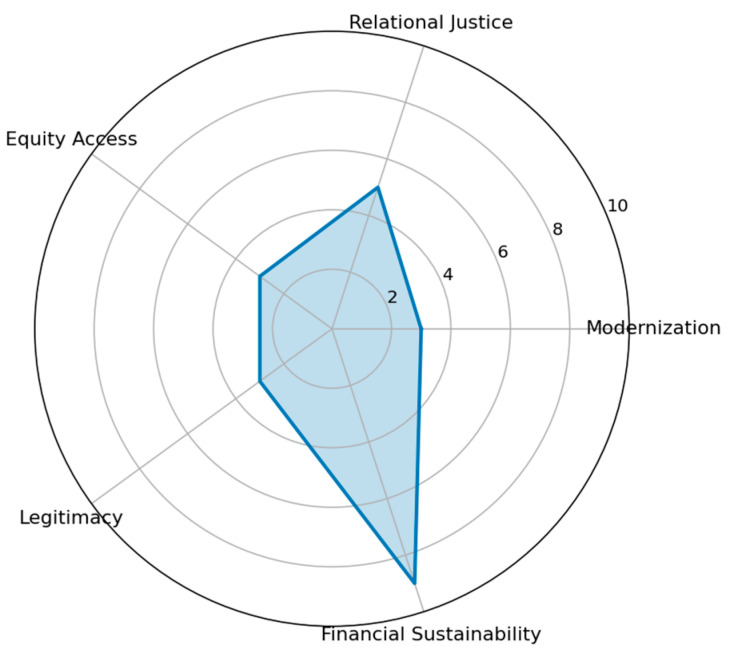
Ethical Profile—Status Quo.

The Status Quo scenario produced the lowest overall weighted governance score (4.1), ranking substantially below both alternative strategies. As illustrated in Figure 6, the radar configuration shows comparatively low values in modernization, equity of access, and legitimacy, while financial sustainability remains the dominant strength. This pattern reflects a fiscally conservative but strategically stagnant governance posture.

The Status Quo achieved the highest financial sustainability score among the three scenarios and generated a modestly negative workload pressure index (−0.7), indicating short-term operational balance. In the absence of major capital investment or workforce expansion, fiscal strain remains minimal and staffing demand does not escalate. While fiscal stability is preserved, the model demonstrates that failure to invest in modernization or workforce capacity perpetuates diagnostic inequality and limits relational gains identified in the qualitative findings. Over time, this configuration risks legitimacy stagnation or erosion, illustrating the governance trade-off between short-term financial prudence and long-term institutional credibility.

**Figure 7:**
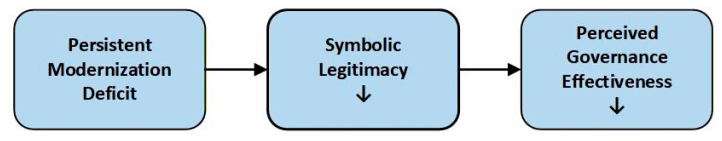
Legitimacy Erosion Under Strategic Inaction.

However, despite short-term fiscal stability, performance across modernization and legitimacy domains remains weak. Without visible investment in technological infrastructure or staffing enhancement, the institution does not generate symbolic or relational gains comparable to the alternative strategies. As depicted conceptually in the systems framework shown in Figure 7, stagnation in modernization produces gradual legitimacy decline. This dynamic directly contrasts with the dominant patient expectations identified in the qualitative interviews, where visible modernization was repeatedly framed as a prerequisite for institutional credibility and equitable care.

Over time, these dynamic risks reinforce perceptions of institutional inferiority and limited responsiveness to community expectations. The absence of visible progress may undermine trust and diminish the hospital’s competitive standing relative to better-equipped facilities.

Accordingly, while fiscally conservative, the Status Quo scenario generates cumulative legitimacy risk. The simulation suggests that short-term financial stability may come at the cost of long-term governance credibility and institutional relevance.

### 4.3. Comparative Ethical Evaluation Table

**Figure 8:**
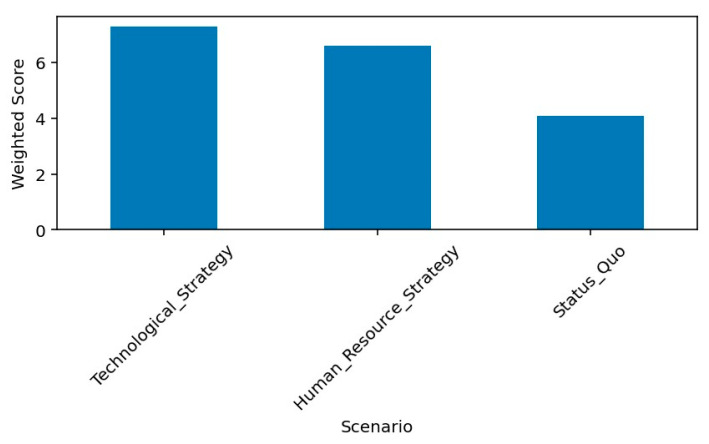
Scenario Comparison—Overall Governance Score.

The comparative results highlight distinct governance trade-offs across the three allocation strategies. As illustrated in Figure 8, the Technological Strategy achieves the highest overall weighted governance score (7.3), followed by the Human Resource Strategy (6.6), while the Status Quo scenario performs substantially lower (4.1). The visual separation between the bars underscores the relative magnitude of difference in aggregate governance performance.

This comparative configuration directly reflects the structured patient expectations identified in the qualitative findings, where modernization, relational justice, and legitimacy emerged as central governance criteria. Technological Strategy maximizes modernization and symbolic legitimacy, producing the highest overall governance score. However, these gains are accompanied by increased workload pressure and reduced financial sustainability, indicating that visible modernization generates operational and fiscal strain unless supported by complementary adjustments.

The Human Resource Strategy achieves the strongest performance in relational justice and operational balance. By expanding staffing capacity, it stabilizes workload dynamics and enhances procedural fairness. Nevertheless, it does not resolve structural diagnostic inequalities, as modernization remains moderate and imaging capacity unchanged.

The Status Quo preserves short-term fiscal stability but produces the weakest legitimacy profile overall. Strategic inaction generates gradual legitimacy erosion despite budgetary equilibrium, underscoring the cumulative risks of non-investment.

Importantly, sensitivity testing confirmed model stability: moderate parameter variation did not alter scenario ranking. This robustness strengthens confidence that the observed trade-offs are structural rather than artifacts of weighting assumptions.

These findings demonstrate that managerial allocation decisions generate multidimensional and cascading governance consequences. Improvements in one domain, such as modernization or relational justice, produce downstream effects in workload dynamics, fiscal stability, and symbolic legitimacy. Therefore, resource allocation in municipal hospitals cannot be evaluated through single-domain performance indicators. Instead, integrated frameworks combining legitimacy theory, distributive and relational justice principles, and systems-dynamics analysis are necessary to capture the full governance implications of strategic decision-making.

## 5. Discussion

In this study, patient perception is conceptualized as a multidimensional evaluation of hospital governance encompassing experiential satisfaction, symbolic legitimacy, distributive fairness, and relational justice. The findings show that patient satisfaction alone is insufficient to capture the governance implications of managerial decision-making in municipal hospitals. Empirical investigations have questioned whether patient satisfaction consistently correlates with favorable clinical outcomes, suggesting that experiential measures may not fully capture institutional performance [20]. The findings of the present study confirm and extend this perspective by demonstrating that patient evaluations in municipal hospitals are structured not only around satisfaction but also around institutional legitimacy, encompassing symbolic and distributive dimensions. While Romanian studies have emphasized experiential evaluations of care quality [4,5], the present analysis shows that patient assessment operates at the level of institutional legitimacy. While consistent with prior Romanian studies, the present findings extend this literature by showing that patient perceptions also incorporate broader expectations related to modernization, equity, and responsiveness. Participants evaluated not only interpersonal care but also visible modernization, distributive fairness, and strategic responsiveness. Satisfaction therefore reflects primarily pragmatic performance, whereas modernization and equity perceptions contribute to symbolic and moral legitimacy [11]. Governance frameworks relying exclusively on satisfaction metrics risk overlooking these broader dimensions of institutional credibility.

The second central thematic axis concerns ethical trade-offs in resource allocation. Resource allocation in structurally constrained municipal hospitals involves unavoidable ethical trade-offs. The simulation illustrates the tension between capital-intensive technological modernization and relational investment in workforce capacity. Technological acquisition strengthens symbolic legitimacy and improves perceived equity of access, yet it increases fiscal exposure and workload pressure. Conversely, workforce expansion enhances relational justice and operational stability but leaves structural modernization gaps unresolved. This dilemma reflects the equity-efficiency tension described in distributive justice and access theory [3] and aligns with ethical conceptions of professional responsibility in care delivery [12]. These findings confirm existing equity-efficiency frameworks and extend them by showing how patients directly perceive these trade-offs. Socio-economic and system-level provision factors have also been shown to significantly influence satisfaction evaluations, reinforcing the structural determinants of perceived quality [21]. In contrast to studies focusing primarily on socio-economic determinants, the present study highlights the role of managerial allocation strategies as a central driver of perceived quality and legitimacy. No allocation strategy is normatively dominant; each redistributes ethical advantages and vulnerabilities across domains.

These trade-offs are intensified in the Romanian context of decentralization and uneven resource distribution [1,2]. The present findings are consistent with this literature, confirming that decentralization and resource constraints intensify governance trade-offs, while further demonstrating how these structural conditions are interpreted at the level of patient perception. Municipal managers must balance fiscal sustainability with visible responsiveness and distributive correction. The findings indicate that allocation strategies do not eliminate governance pressures but reallocate them. Ethical governance therefore requires explicit recognition of opportunity costs and distributive consequences rather than reliance on isolated performance indicators.

The integration of systems thinking further reveals that governance outcomes are dynamically contingent. Technological modernization generates reinforcing effects: in-creased modernization strengthens legitimacy, which may attract greater patient inflow and elevate workload pressure. Without proportional workforce adjustment, initial symbolic gains may undermine operational stability. Similarly, status quo preservation may secure short-term fiscal balance but gradually erode legitimacy through perceived stagnation. These patterns demonstrate that governance effects are non-linear and evolve through feedback processes. Linear evaluation models are therefore insufficient for anticipating the long-term consequences of strategic allocation decisions [8]. These results extend prior system-based analyses by illustrating how feedback mechanisms are not only organizational but also perceptual, influencing legitimacy formation through patient experience over time.

The study carries several policy implications for Romanian municipal hospitals. First, strategic prioritization should incorporate legitimacy effects alongside financial and operational indicators. Visible modernization can yield substantial symbolic gains but must be accompanied by workforce planning to prevent workload amplification. Second, budget planning under constraint should balance capital and relational investments. A sequenced or hybrid approach, combining targeted staffing reinforcement with phased technological upgrading, may mitigate extreme trade-offs identified in the simulation. Third, governance models should become explicitly legitimacy-sensitive. Incorporating patient expectations into strategic evaluation strengthens transparency and participatory responsiveness, reinforcing institutional credibility even under fiscal limitation.

In transitional systems marked by structural underinvestment, municipal managers operate within constrained decision spaces. By integrating ethical analysis, legitimacy theory, and systems modeling, this study provides a structured framework for evaluating allocation dilemmas beyond satisfaction metrics alone. Legitimacy-aware and systems-informed governance may enhance both sustainability and public trust. These findings extend prior qualitative evidence by integrating patient-derived expectations into a structured simulation framework, thereby moving from descriptive analysis toward a predictive and governance-oriented model of hospital decision-making [6].

## 6. Limitations

This study has several limitations. First, it is based on qualitative data from a single Romanian municipal hospital. Although the findings reflect broader structural patterns [1,2], the findings do not constitute a statistically representative sample of Romanian municipal hospitals or the national healthcare system. The study therefore seeks analytic rather than statistical generalization. To support the simulation component, a synthetic population of 1000 simulated agents was subsequently generated based on the demographic and thematic distributions observed in the 12 participants. The simulation therefore enhances internal analytical coherence but does not alter the underlying qualitative scope.

Second, the simulation model is conceptual rather than econometric. Parameter weights were derived from thematic salience rather than large-scale quantitative data. The model is therefore designed as a structured comparative tool for examining governance trade-offs rather than as a predictive instrument.

Third, the synthetic population generated for simulation purposes was constructed to preserve observed demographic and thematic distribution from the qualitative sample. While this approach enhances internal model coherence, it does not substitute for large-scale empirical validation. The synthetic cohort serves a parameterization function rather than a representational one.

Fourth, participation was consultative. Although patient perspectives informed the modeling framework, patients were not directly involved in co-designing scenarios or validating outputs. Future research could extend participatory depth through deliberative or co-production approaches.

Finally, broader macroeconomic and national reimbursement dynamics were not explicitly modeled. Incorporating multi-level fiscal interactions may strengthen future analysis of municipal hospital governance under structural constraint.

Despite these limitations, the study advances an integrated framework linking resource allocation ethics, organizational legitimacy, participatory governance, and systems thinking. This approach offers a transferable analytical model for examining allocation dilemmas in non-resource-constrained municipal hospitals.

## 7. Conclusions

Municipal hospitals operating within transitional health systems face persistent structural constraints that render resource allocation decisions ethically complex and strategically consequential. In the Romanian context of decentralization and uneven financial reinforcement, managerial choices regarding technological modernization, workforce expansion, or fiscal preservation extend beyond operational efficiency and shape broader perceptions of institutional legitimacy.

This study shows that patient evaluation cannot be captured through satisfaction metrics alone. Qualitative findings reveal that patients interpret visible modernization, distributive fairness, and relational justice as indicators of institutional seriousness and credibility. By integrating resource allocation ethics, organizational legitimacy theory, participatory governance principles, and systems thinking, the study reframes patient perception as a legitimacy-sensitive construct embedded within governance dynamics.

The conceptual simulation model illustrates that allocation strategies generate non-linear and interdependent effects. Technological investment enhances symbolic legitimacy but may intensify workload and fiscal exposure. Workforce expansion strengthens relational justice and operational stability but does not fully address structural modernization disparities. Status quo preservation may protect short-term fiscal balance yet risks gradual erosion of institutional credibility. These findings underscore that allocation strategies redistribute governance pressures rather than eliminate them.

Methodologically, the study contributes a novel approach by translating qualitative patient expectations into structured governance parameters. Rather than relying solely on descriptive satisfaction analysis, the research defines ethical and legitimacy dimensions within a comparative simulation framework. This approach enables systematic evaluation of trade-offs under scarcity without presupposing a single optimal strategy.

For Romanian municipal hospitals, the findings suggest that legitimacy-sensitive and systems-informed governance may enhance both sustainability and public trust. Strategic prioritization should account not only for financial constraints but also for the symbolic and distributive signals conveyed by managerial decisions. Incorporating patient expectations into structured allocation analysis strengthens transparency and aligns strategic planning with community-defined priorities.

In resource-constrained transitional systems, hospital governance requires balancing equity, efficiency, relational justice, and institutional credibility within complex feedback environments. By integrating ethical theory with dynamic modeling, this study provides a transferable analytical framework for examining allocation dilemmas in municipal healthcare institutions.

## Figures and Tables

**Table 1:** Participant Demographic Characteristics.

Characteristic	Value
Age (Mean ± SD)	59.25 ± 9.39
Age (Min–Max)	44–76
**Age Groups**	
41–60 Years	50.00%
>60 years	50.00%
**Sex**	
Male	66.70%
Female	33.30%
**Education level**	
Primary School	25.00%
Middle School	8.30%
Secondary School	58.30%
Post-Secondary School	8.30%

**Table 2:** Code-to-variable Mapping Framework.

Example Coded Speech	Initial Code	Thematic Cluster	Derived Conceptual Variable	Modeled Outcome in Simulation
“We need a CT here. It’s not normal to send patients to another city.”	Diagnostic referral burden	Technological modernization	Institutional Modernization Index (Mt)	↑ Equity of Access ↑ Legitimacy
“Without modern equipment, people don’t trust the hospital”	Equipment as credibility signal	Technological modernization	Perceived Modernization Signal	↑ Symbolic Legitimacy
“There are too few doctors. They are always overwhelmed”	Staff shortage	Human resource capacity	Workforce Capacity Index	↑ Relational Justice ↓ Workload pressure
“You wait a long time before someone explains anything”	Communication delay	Human resource capacity	Staffing responsiveness	↑ Patient Satisfaction
“The building needs renovation; it looks old.”	Facility deterioration	Infrastructure modernization	Infrastructure Quality Index	↑ Institutional Image ↑ Symbolic Legitimacy
“Clean room make you feel safer.”	Environmental safety perception	Infrastructure modernization	Environmental Confidence Indicator	↑ Perceived Quality
“Doctors explain things clearly, and that matters.”	Clear explanation	Communication/relational care	Relational Justice	↑ Patient Satisfaction ↑ Legitimacy
“Respect and empathy are very important.”	Dignity expectation	Communication/relational care	Relational Justice Index	↑ Trust ↑ Moral Legitimacy
“If the hospital had proper equipment, it would be equal to bigger cities.”	Perceived inequality	Technological modernization	Equity of Access	↑ Distributive Legitimacy

↑: increasing, ↓: decreasing.

**Table 3:** Governance variables and baseline parameter weights.

Parameter	Governance Domain	Weight
θ	Perceived Legitimacy (P)	0.10
λ	Patient Satisfaction	0.00
γ	Equity of Access (E)	0.20
δ	Financial Sustainability (F)	0.15
β	Institutional Modernization (M)	0.25
α	Relational Justice (R)	0.30
Total		1.00

**Table 4:** Definitions of governance variables and ethical outcome.

Variable	Definition	Reference
Equity of Access	Degree to which patients can obtain necessary diagnostic and therapeutic services without structural barriers.	[3]
Relational Justice	Perceived fairness, respect, empathy, and quality of interpersonal interaction in care delivery.	[12]
Institutional Modernization	Level of technological and infrastructural advancement, including symbolic signaling of institutional progress.	[4,5]
Perceived Legitimacy	Aggregate patient judgment of institutional credibility, responsiveness, and alignment with expectations.	[11]
Financial Sustainability	Capacity of the hospital to maintain operations without generating long-term fiscal imbalance.	[2]
Ethical profile	Composite evaluation of governance performance across distributive, relational, symbolic, and financial domains derived from simulation outputs.	This study

## Data Availability

Dataset available on request from the authors.
